# Impact of Pregnancy on Zonisamide Pharmacokinetics in Rabbits

**DOI:** 10.1155/2013/140327

**Published:** 2013-12-18

**Authors:** Kamal M. Matar

**Affiliations:** Department of Pharmacology and Therapeutics, Faculty of Pharmacy, Kuwait University, P.O. Box 24923, 13110 Safat, Kuwait

## Abstract

Pregnancy is associated with various physiological changes which may lead to significant alterations in the pharmacokinetics of many drugs. The present study was aimed to investigate the potential effects of pregnancy on the pharmacokinetic profile of zonisamide (ZNM) in the rabbit. Seven female rabbits were used in this study. The pregnant and nonpregnant rabbits received ZNM orally at a dose of 10 mg/kg and blood samples were collected from the animals just before receiving the drug and then serially for up to 24 h. The plasma samples were analyzed using tandem mass spectrometric method. Following a single oral dose of ZNM to the rabbits, the mean values of ZNM plasma concentrations at different times were consistently low in pregnant compared to nonpregnant rabbits. The mean values of ZNM's *C*_max_ and AUC_0−*∞*_ were significantly (*P* < 0.05) decreased, whereas the CL/F exhibited substantial increase (*P* < 0.05) in pregnant compared to nonpregnant rabbits. *T*_max_, *t*_1/2abs_, *t*_1/2el_, MRT, and Vd/F showed no significant differences between the two groups. The present study demonstrates that pregnancy decreased ZNM plasma concentrations in rabbits and that the decrease could be due to decreased extent of gastrointestinal absorption, induced hepatic metabolism, or enhanced renal elimination of the drug.

## 1. Introduction

Zonisamide (ZNM) is a 1,2-benzisoxazole-3-methanesulfonamide, structurally unrelated to other antiepileptic drugs (AEDs) in clinical practice. The precise mechanism of ZNM by which it exerts its antiepileptic effects is not yet known. However, it has been proposed that ZNM stabilizes neuronal membranes by blocking voltage-sensitive Na^+^ channels and also inhibiting low-threshold T-type Ca^2+^ channels [1]. It is used as an adjunctive therapy in the treatment of partial seizures (with or without generalization) in adults [1]. Following oral administration, ZNM is almost completely absorbed from the gastrointestinal tract, irrespective of food intake reaching maximal plasma concentrations within 2–5 h under fasting conditions and 4–6 h with food [2, 3]. It is predominantly metabolized in the liver with subsequent elimination by the kidney. About 50% of ZNM is metabolized by cytochrome P450 (CYP), CYP3A4 isoenzyme. However, CYP2C19 and CYP3A5 may also contribute [4]. In addition, acetylation accounts for 20% of ZNM metabolism [3]. Approximately 30% of the parent drug is excreted unchanged in the urine [3]. Although ZNM is only 40–60% plasma protein bound, it is extensively bound to erythrocytes [2, 3]. The therapeutic level of ZNM is usually in the range of 10–40 *μ*g/mL [5].

Epilepsy is the most common neurologic condition that requires continuous management during pregnancy and AEDs are associated with increased risk of teratogenicity in this population [6]. The management of epilepsy during pregnancy requires a balance between minimizing fetal exposure to AEDs and maintaining seizure control [7]. Although it has been reported that ZNM is teratogenic in animals, very limited data are available on the extent of teratogenicity in humans and further clinical investigations are warranted to confirm the preliminary findings [8, 9].

Pregnancy is associated with various physiological changes which may lead to considerable variations in the disposition of many drugs including AEDs [10]. During pregnancy, the plasma levels of AEDs tend to decrease as pregnancy progresses, with potential consequences of increasing seizure frequency [10, 11]. Understanding the pharmacokinetic alterations of AEDs during pregnancy is clinically important as the decrease in the plasma concentrations of these drugs has been associated with deterioration of seizures control and this could adversely affect the pregnancy outcomes. Therefore, therapeutic drug monitoring for these drugs might be of value during pregnancy [10]. There have been no systematic investigations on the potential influence of pregnancy on ZNM pharmacokinetics and the data are limited. There exists only one case report on ZNM plasma levels during pregnancy [5]. However, the report did not explore the underlying mechanisms responsible for the decrease in ZNM plasma levels.

The objective of the present study was to investigate the potential effects of pregnancy on the pharmacokinetic profile of ZNM in the rabbit.

## 2. Materials and Methods

### 2.1. Chemicals and Reagents

Zonisamide pure standard was purchased from Sigma-Aldrich (St. Louis, MO, USA) and the internal standard (IS), [^2^H_4_, ^15^N]-zonisamide, was purchased from Alsachim Co. (Strasbourg, France). Zonegran (100 mg capsule), the commercial formulation of ZNM (Eisai Ltd, Hertfordshire, UK), was purchased from a drug store. Water was purified using a Milli-Q water device (Millipore, Bedford, MA, USA). All other chemicals and reagents were of analytical grade and solvents were of HPLC grade.

### 2.2. Ethical Approval

Ethical approval for conducting this study was provided by the local Experimental Animal Resource Center Ethics Committee, Health Sciences Center, Kuwait University. The study was conducted in compliance with the Helsinki Declaration for ethical principles of medical research.

### 2.3. Blood Sampling

Seven female New Zealand white rabbits weighing between 3.5 and 5 kg were used in this study. The animals were acclimatized in a separate room, under controlled lighting and heating conditions, in Experimental Animal Resources Center for 1 week before inclusion in the study. The rabbits were maintained on food and water *ad libitum*. Each animal was physically examined and was considered healthy before inclusion in the study. The study was conducted in a crossover design with a washout period of six weeks. The same animal was used as a control (nonpregnant) for itself. Vulvas of the rabbits were thoroughly examined for any potential infection before mating. The receptive female (vulva was red and dilated) was cohabited with the male breeder and they were kept together in the cage for ~2–4 h before they were separated. The mating day was considered as the day one of pregnancy. Two weeks later, pregnancy was verified by palpation of the abdomen, changes in the body weight, and pulling off the animal's fur near term. The length of gestational period in rabbits normally varies between 30–35 days. In the pregnant rabbit, the study was performed on day 28 of gestation by administering a single dose of ZNM orally (by gavage) at a dose of 10 mg/kg in a freshly prepared suspension (20 mg/mL ZNM in 0.5% CMC). Blood samples (~0.5 mL) were serially collected into preheparinized (10 *μ*L) plastic centrifuge tubes (1.5 mL). The blood samples were collected through a cannula inserted into the marginal ear vein just before dosing and at 0.5, 1.0, 1.5, 2, 4, 6, 8, 12, 20, and 24 h following ZNM administration. The blood samples were immediately centrifuged at 9,000 ×g for 10 min and then plasma samples were separated and stored at −80°C pending analysis.

### 2.4. Analytical Procedure

#### 2.4.1. Instrumentation

A liquid chromatographic system, Alliance 2695, consisted of a solvent delivery system, and an autosampler (Waters Assoc., Milford, MA, USA) was used. Chromatographic separation of the analytes was achieved on Symmetry C_18_ column (5 *μ*m, 3.9 × 50 mm) equipped with a precolumn filter of the same packing material. The mobile phase consisted of acetonitrile-0.1% triethylamine (80 : 20, v/v; pH = 9.9) and delivered at a flow rate of 0.2 mL/min to a negative electrospray ionization interface (ESI−) of triple quadrupole mass spectrometer (Quattro LC, Micromass, Manchester, UK). Tuning parameters of MS were optimized by direct infusion of solutions of ZNM and IS in the mobile phase into the ionization probe at a flow rate of 10 *μ*L/min using Hamilton syringe. The ion source and desolvation temperatures were set at 150°C and 350°C, respectively. The capillary voltage was adjusted at 3.18 kV, cone voltage at 20 V, collision energy at 12 eV, and collision gas pressure at <1.0*e* ^−4^ mbar. The MRM transitions at *m/z* 211 > 118.8 and 216 > 122.8 were selected for quantification of ZNM and IS, respectively. Data acquisition, handling, and system control were performed by MassLynx Software (Version 4.1, Micromass, Manchester, UK).

#### 2.4.2. Preparation of Calibration Standards and Quality Control Samples

Stock solutions of ZNM and the internal standard ([^2^H_4_, ^15^N]-zonisamide) were prepared by dissolving the compounds in methanol to yield 1.0 mg/mL solutions. Aliquots of ZNM and the IS stock solutions were further diluted with methanol to yield the working standard solutions of 500 *μ*g/mL and 100 *μ*g/mL, respectively. The calibration standards of ZNM at concentrations of 0.5, 1.0, 5, 10, 15, 20, 30, and 50 *μ*g/mL were prepared by spiking drug-free rabbit plasma with ZNM standard solution. Similarly, quality control (QC) samples at concentrations of 2, 12, 25, and 40 *μ*g/mL were prepared by spiking drug-free rabbit plasma with ZNM solution. The spiked plasma samples were aliquoted (200 *μ*L) into Eppendorf polypropylene tubes and kept frozen at −80°C pending analysis.

#### 2.4.3. Sample Preparation

Prior to assay, frozen rabbit plasma samples, including calibrators or QC samples, were thawed at ambient temperature. A 100 *μ*L aliquot of each plasma sample was transferred to a 1.5 mL Eppendorf tube and then 20 *μ*L of IS (100 *μ*g/mL) was added and vortex-mixed for 30 sec. To each tube, 20 *μ*L of ammonium acetate (1.0 mM) and 1.0 mL of diethylether were added and vortex-mixed for 30 sec. The tube was centrifuged at 9000 ×g for 10 min, the organic layer was separated and evaporated under stream of purified N_2_ gas and then reconstituted with 150 *μ*L of mobile phase. A 10 *μ*L of this sample was then injected into the LC-MS system for analysis. The assay method was fully validated for linearity, accuracy, precision, selectivity, stability, and matrix effect according to the standard guidelines [12].

### 2.5. Pharmacokinetics and Statistical Analyses

ZNM pharmacokinetic parameters were estimated by standard noncompartmental methods using Kinetica software, version 5.1 (Thermo Fisher Scientific, USA). The maximum plasma concentration (*C*_max⁡_) and time needed to attain this concentration (*T*_max⁡_) were directly obtained from the drug plasma profiles; the drug plasma elimination half-life (*t*_1/2el_) values were calculated as ln⁡2/*k*_el_, where *k*_el_ is the elimination rate constant. The absorption half-life (*t*_1/2abs_) was determined from the plasma concentration-time profiles employing method of residuals (feathering technique). The area under the plasma concentration-time curves (AUC_0–*t*_) was calculated from the measured data points from time zero to time of last quantifiable concentration by the linear trapezoidal rule and the area under the plasma concentration-time curves extrapolated to time infinity (AUC_0–*∞*_) was calculated using the equation AUC_0–*∞*_ = AUC_0–*t*_ + C*/*k*_el_, where C* is the last quantifiable drug plasma concentration. The mean residence time (MRT) was calculated as AUMC_0–*∞*_/AUC_0–*∞*_, where AUMC_0–*∞*_ is the area under the first moment of plasma concentration-time curve from time zero to time infinity. Oral body clearance (CL/F) was calculated as CL/F = Dose/AUC_0–*∞*_ and the volume of distribution (Vd/F) was calculated as Vd/F = (CL/F)/*k*_el_. The pharmacokinetic parameters were presented as mean ± SD. Differences between the pharmacokinetic parameters of ZNM among the groups were considered statistically significant if *P* < 0.05 using Wilcoxon matched-pair signed-rank test (two-tailed). The statistical analysis was performed using the statistical package for social sciences (SPSS) software, version 20 (SPSS Inc., Chicago, IL, USA).

## 3. Results

The tandem mass spectrometric (LC-MS/MS) assay method was developed, fully validated, and employed for quantification of ZNM plasma samples. The linear range of the method was 0.5–50 *μ*g/mL (*r* ^2^ > 0.99) and the lower limit of quantification was 0.5 *μ*g/mL. The intra- and interrun precisions, as measured by relative standard deviations (RSD,%), of the method were less than 7%.

**Figure 1:**
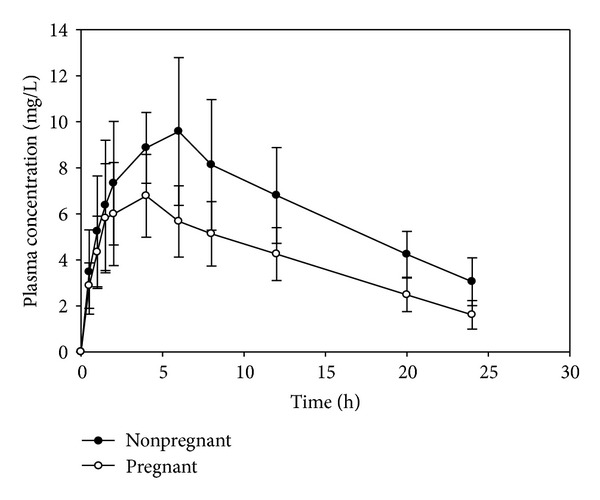
Mean (±SD) plasma concentration-time profile following an oral dose of 10 mg/kg zonisamide to pregnant and nonpregnant rabbits; *n* = 7.

The mean (±SD) plasma concentration-time profile following a single oral dose of ZNM (10 mg/kg) administered to pregnant and nonpregnant (control) rabbits is depicted in Figure 1 and the mean (±SD) pharmacokinetic parameters of ZNM in the two groups are presented in Table 1. Figure 1 and Table 1 demonstrated substantial interanimal variability among the two groups. Statistical comparison of the mean values of ZNM pharmacokinetic parameters resulted in a significant (*P* < 0.05) decrease in *C*_max⁡_, AUC_0–*t*_, and AUC_0–*∞*_ in pregnant compared to nonpregnant rabbits. In addition, the oral clearance (CL/F) was significantly (*P* < 0.05) increased in pregnant rabbits in comparison with nonpregnant group. The other pharmacokinetic parameters, involving *T*_max⁡_, *t*_1/2abs_, *t*_1/2el_, MRT, and the volume of distribution (Vd/F), were not significantly different in both pregnant and nonpregnant rabbits (*P* > 0.05). However, the mean value of Vd/F was increased by about 35% in pregnant compared to nonpregnant rabbits.

## 4. Discussion

The present study was aimed at investigating the influence of pregnancy on the pharmacokinetic profile of ZNM in rabbits because well-studied, detailed pharmacokinetic investigations involving alterations and time course of alterations are lacking and knowledge of pharmacokinetic profile of ZNM during pregnancy is important in order to optimize drug therapy. The present study was performed at late pregnancy stage (28-29 days' gestation) because at this period, the physiological alterations as a consequence of pregnancy are expected to be pronounced which would potentially affect the pharmacokinetics of ZNM.

To the best of my knowledge, this is the first attempt to investigate the potential effects of pregnancy on the pharmacokinetics of ZNM. The present data demonstrated that pregnancy significantly (*P* < 0.05) modified the pharmacokinetic parameters (*C*_max⁡_, AUC_0–*t*_, AUC_0–*∞*_, and CL/F) of ZNM in rabbits. However, the other pharmacokinetic parameters (*T*_max⁡_, *t*_1/2abs_, *t*_1/2el_, MRT, and Vd) demonstrated no significant alterations by pregnancy (*P* > 0.05), Table 1. Although the mean Vd/F was increased by about 35% in pregnant compared to nonpregnant rabbits, it did not reach the level of significance.

There has been only one published case report on ZNM concentrations during pregnancy [5]. In that report, ZNM plasma levels were followed regularly in a pregnant patient starting from the fifth gestational week until the last week of pregnancy. The patient was on ZNM monotherapy at a daily dose of 200 mg until week 29 and the plasma levels of the drug were in the range of 7.5 to 10.1 *μ*g/mL (from fifth week to week 22) and 4.4 *μ*g/mL in week 27. Owing to low ZNM plasma levels, the daily dose was increased to 300 mg at week 29 and maintained at this level until delivery. The case report did not investigate the pharmacokinetic profile that could elucidate the underlying mechanisms responsible for reduced ZNM plasma concentrations during pregnancy because the study was based on sparse concentration measurements of only one pregnant patient. However, the investigators have recommended utilization of therapeutic monitoring of ZNM levels throughout pregnancy.

Several mechanisms have been proposed to explain the decrease in the plasma concentrations of AEDs during pregnancy. These include reduced gastrointestinal drug absorption, increased volume of distribution, altered drug protein binding, enhanced metabolism, and increased drug renal clearance [10, 13]. The findings of the present study demonstrated that ZNM plasma concentrations were consistently low in pregnant compared to nonpregnant rabbits and the decline in the plasma levels demonstrates that the present investigation supports and concurs with the observation of the previous case report [5], Figure 1. However, the advantage of the present investigation over the previous case report is an endeavor to explain the underlying mechanisms causing the decline in ZNM plasma levels during pregnancy. The decline in ZNM plasma levels during pregnancy in the present study is most likely caused by a combination of several mechanisms. One of these mechanisms could be a reduced gastrointestinal absorption of ZNM. As shown in Table 1, both *C*_max⁡_ and AUC_0−*∞*_ were significantly (*P* < 0.05) decreased in pregnant rabbits in comparison with nonpregnant group leading to a reduction in the extent of ZNM absorption, whereas the rate of ZNM absorption was not significantly altered, as demonstrated by the unaltered *T*_max⁡_,   *t*_1/2abs_, and MRT, values in both groups (*P* > 0.05) [10]. Other mechanisms that could be attributed to the reduced ZNM plasma levels during pregnancy could be enhanced ZNM elimination as reflected by a marked increase (*P* < 0.05) in ZNM oral clearance in pregnant compared to nonpregnant rabbits. Pregnancy is known to induce changes in the hepatic metabolism of many drugs by affecting the enzymes responsible for the drug metabolism such as CYP450 and UGT. Activity of enzymes involving CYP3A4, CYP2D6, CYP2C9, UGT1A4, and UGT2B7 is increased during pregnancy, whereas the activity of CYP1A2 and CYP2C19 is decreased [13–15]. ZNM is primarily metabolized by CYP3A4 isoenzymes, where their activities are known to be induced during pregnancy, which in turn may contribute to the low plasma concentrations of ZNM during pregnancy in rabbits. In addition, a significant (*P* < 0.05) increase in ZNM oral clearance could explain the reduced plasma levels of the drug owing to enhanced renal blood flow and glomerular filtration rate (GFR) during pregnancy [16]. In pregnancy, however, the renal plasma flow increases leading to an increase in the GFR (up to 50–80%). This results in an increase of 20–65% in the renal clearance of many drugs [13, 16]. It has been reported that 30% of ZNM is eliminated as unchanged in the urine [3]. So, during pregnancy ZNM renal clearance is expected to be enhanced, and this may contribute to low ZNM plasma concentrations in the present study. Moreover, pregnancy is associated with an increase in the volume of distribution of many drugs due to an increase in the plasma volume and a decrease in plasma protein binding. Although the mean value of Vd/F of ZNM has been increased by about 35% in pregnant compared to nonpregnant rabbits, this effect is unlikely to influence ZNM plasma levels because the drug is not significantly bound to plasma proteins (40–60%) [3, 10].

The findings of the present study indicate that the pharmacokinetic profile of ZNM in rabbits is altered in late gestational period. Although limited by a relatively small sample size and a large interanimal variability in ZNM pharmacokinetics observed within the same group, the present study suggests that the decline in ZNM plasma levels during pregnancy is most likely caused by decreased extent of gastrointestinal absorption, induced enzyme induction, and enhanced renal elimination of the drug. Further studies on a large number of appropriate subjects using single dose as well as multiple doses at various stages of pregnancy are warranted. Extrapolation of the findings of the present study to humans suggests consideration of therapeutic drug monitoring for ZNM during pregnancy and understanding the pharmacokinetic alterations of ZNM during pregnancy is clinically important in an attempt to optimize drug therapy since these alterations may potentially affect the seizure control.

## Figures and Tables

**Table 1:** Mean (±SD) pharmacokinetic parameters of zonisamide in pregnant and nonpregnant rabbits.

Parameter	Nonpregnant	Pregnant	*P* value^a^
*T*_max⁡_ (h)	4.33 ± 1.97	3.33 ± 1.03	0.344
*C*_max⁡_ (*μ*g/mL)	10.24 ± 2.90	7.21 ± 1.84	0.036
*t*_1/2abs_ (h)	1.86 ± 1.62	0.94 ± 0.36	0.313
*t*_1/2el_ (h)	13.72 ± 5.11	10.84 ± 3.43	0.313
MRT (h)	20.92 ± 6.44	16.38 ± 5.17	0.156
AUC_0–*t*_ (*μ*g·h/mL)	150.35 ± 36.70	97.88 ± 24.87	0.031
AUC_0–*∞*_ (*μ*g·h/mL)	213.86 ± 45.59	124.10 ± 31.60	0.031
Vd/F (L/kg)	0.98 ± 0.51	1.32 ± 0.59	0.313
CL/F (mL/min/kg)	0.82 ± 0.21	1.40 ± 0.27	0.031

*T*_max⁡_: time needed to reach maximum plasma concentration; *C*_max⁡_: maximum plasma concentration; *t*_1/2abs_: absorption half-life; *t*_1/2el_: elimination half-life; MRT: mean residence time; AUC_0–*t*_: area under the plasma concentration-time curve from time 0 to time of last quantifiable concentration; AUC_0–*∞*_: area under the plasma concentration-time curve from time 0 to infinity; Vd/F: volume of distribution; CL/F: oral clearance.

^a^Statistically significant if *P* < 0.05, using Wilcoxon matched-pair signed-rank test; *n* = 7.
